# Phytochemical directed synthesis of flower-like ZnO nanostructures from almond peel extract for sunlight-activated congo red degradation

**DOI:** 10.1371/journal.pone.0345449

**Published:** 2026-04-30

**Authors:** Sujesh Sudarsan, Jonas Van Caeneghem, Ramesh Vinayagam, Raja Selvaraj

**Affiliations:** Manipal Institute of Technology, Manipal Academy of Higher Education, Manipal, India; Sri Vidya Mandir Arts & Science College (Autonomous), INDIA

## Abstract

This study reports a low-temperature green route for producing sunlight-active zinc oxide (ZnO) nanoparticles using almond peel extract as the sole complexing and stabilizing medium and evaluates their performance toward Congo red (CR) removal from water. UV-visible analysis of the synthesized material shows a near UV absorption edge at 373 nm with an optical band gap energy of 3.32 eV. The photocatalyst possesses a mesoporous texture with a specific surface area of 31.89 m^2^/g and a mean pore diameter of 9.8 nm. FESEM reveals hierarchical flower-like assemblies composed of radially oriented ZnO rods, supported by EDS, confirming Zn and O as the dominant elements. XRD verifies phase-pure hexagonal wurtzite ZnO structures with a mean crystallite size of 39.24 nm. FTIR and XPS confirm ZnO formation and a phytochemical-derived surface overlayer containing hydroxyl, carbonyl, and aromatic functionalities, together with surface hydroxyls and chemisorbed oxygen. Photoluminescence indicates near-band-edge emission and defect-related visible bands consistent with oxygen vacancy and zinc-related trap states. Under natural sunlight (CR: 25 mg/L; catalyst dosage: 5–15 mg/100 mL; irradiation time 270 min), the material achieved 51.61 to 82.21% degradation and followed pseudo-first-order behaviour. Scavenger studies revealed that O_2_•^−^ radicals are the dominant reactive species in dye degradation, with photogenerated h^+^ playing a secondary role and •OH contributing minimally. Also, the ZnO nanoparticles exhibited good reusability, retaining over 83.64% of their initial photocatalytic activity after four cycles under sunlight irradiation. Thus, almond peel-derived ZnO offers a simple strategy to valorize an agricultural residue into a sunlight-responsive photocatalyst for treating dye-contaminated wastewater.

## 1. Introduction

Synthetic dyes are commonly used in leather, paper, textile, and printing industries, and a considerable fraction of the applied colour is discharged into wastewater without adequate treatment. Among the various dye classes, azo dyes form the largest group and are of particular concern because their aromatic azo linkages are resistant to biological degradation and can generate toxic aromatic amines during transformation [1]. Congo Red (CR), a benzidine-based diazo dye, is commonly employed as a colourant and is often selected as a model pollutant for recalcitrant anionic dyes in laboratory studies [2]. Its high colour intensity, structural stability, and resistance to biodegradation allow it to persist in aquatic bodies, where it reduces light penetration, interferes with photosynthesis, and exerts mutagenic and carcinogenic effects [3]. These risks motivate the development of treatment strategies that achieve not only decolourization but also substantial mineralization of CR in wastewater.

Physical, chemical, and biological techniques like coagulation and flocculation, adsorption, ozonation, membrane separation, electrochemical oxidation, and microbial degradation have been applied to dye-contaminated effluents [4]. However, many of these approaches suffer from incomplete degradation of aromatic structures, secondary sludge generation, and high operational cost [5,6]. Advanced oxidation process (AOP) that produces reactive oxygen species (ROS) *in situ* have therefore attracted attention because they can transform complex dye molecules into smaller and less harmful products. Within this group, heterogeneous photocatalysis using semiconductor materials is especially attractive, as it can operate in aqueous media under mild conditions and, in principle, harness sunlight as the energy source [7]. In this context, nanotechnology has emerged as a vital field for environmental remediation, offering the ability to synthesize materials with exceptionally high surface-to-volume ratios and tunable electronic properties. Among various nanomaterials, metal oxide nanoparticles are particularly advantageous due to their diverse morphology, chemical stability and high catalytic activity [8,9].

Zinc oxide (ZnO) is one among the widely studied photocatalysts for dye degradation because it combines a wide band gap in the ultraviolet (UV) region with strong oxidizing ability, good electron mobility, and relative abundance [10]. It can be prepared by simple chemical precipitation or sol-gel routes and remains stable in many wastewater environments [11,12]. At the same time, pristine ZnO absorbs mainly in the UV region, which represents only a small fraction of the solar spectrum, and rapid recombination of photogenerated electrons and holes limits its quantum efficiency [13]. These limitations have motivated strategies to improve the photocatalytic potential of ZnO toward the degradation of recalcitrant dyes.

Conventionally, ZnO nanoparticles are synthesized via physical and chemical routes, including laser ablation, hydrothermal, and sol-gel methods [14]. Although these techniques offer precise control over particle morphology, they often involve high energy consumption, specialized equipment, or the use of toxic reducing and stabilizing agents [15]. To reduce the environmental footprint of ZnO synthesis, while producing defect-rich, surface functionalized photocatalysts, plant-mediated routes use phytochemicals as complexing, reducing, and capping agents for the growing inorganic phase [16]. The resulting metal oxide particles typically carry an organic shell-derived from the extract, which can influence surface charge, introduce additional functional groups, and tune the defect landscape [17]. For example, Alzahrani *et al.* reported that *Haloxylon* and *Calligonum* extracts stabilize ZnO with pronounced O-H, C-O and COO bands and phytochemical-derived surface defects that enhance charge separation and ROS generation during MB degradation [18].

Studies on green ZnO systems prepared from different plant parts for dye removal have reported encouraging decolourization efficiencies [15,19], but these materials still face important limitations. Many reported green ZnO photocatalysts for CR are evaluated mainly under UV lamps, whereas studies under well-characterized natural sunlight remain relatively scarce, even though solar operation is crucial for practical application [20,21]. The sustainability of plant-mediated ZnO synthesis is also constrained by frequent use of high-temperature (400–800 ℃) calcination [22] and by organic solvents during extraction or washing [21], which increases energy demand and can remove beneficial surface organics that would otherwise tune defect structure and facilitate interfacial charge transfer [23]. These gaps motivate water-based, low-temperature green routes that use readily available biomass extracts as benign capping agents, while still yielding ZnO with defect structures and surface chemistries optimized for sunlight-driven photocatalysis.

Within the broader context of biomass utilization, almond processing generates substantial quantities of by-products such as hulls and brown skins that are often discarded [24]. Almond skins, in particular, contain high levels of phenolic acids, flavonoids and tannins with strong antioxidant activity [25]. These phenolic-rich residues therefore represent an attractive yet underutilized source of complexing and stabilizing agents for green synthesis of ZnO nanomaterials. Existing studies on almond-derived biomass have mainly focused on phenolic recovery [26], lignin-based particle synthesis [27], nickel oxide (NiO) nanoparticle fabrication for photocatalysis [28], and carbonaceous adsorbents for dye removal [29,30]. In contrast, the use of aqueous almond peel extract as the sole medium to prepare ZnO photocatalysts for sunlight-driven CR degradation has received far less attention.

The present work synthesizes flower-like ZnO nanoparticles using almond peel extract as the sole reducing, and stabilizing medium, without organic solvents or high-temperature calcination. The material is examined using various characterization methods to establish its structural, surface, and electronic characteristics. The photocatalytic activity of the almond peel-derived ZnO toward CR degradation is evaluated under natural sunlight at different catalyst loadings, and the kinetic behaviour is interpreted using a pseudo-first-order model.

## 2. Materials and methodologies

### 2.1. Materials

Zinc acetate dihydrate (Merck, India) and sodium hydroxide pellets (Merck, India) were employed as the zinc precursor and precipitating agent, respectively. Congo Red (CR, analytical grade, HiMedia Laboratories, India) served as the model azo dye. Fresh almonds were purchased from a local merchant in Manipal, Karnataka, India, soaked overnight in distilled water to soften the kernels, then peeled to obtain the brown skins. The recovered almond peels were rinsed many times with distilled water, air dried at room temperature, and stored in sealed containers for later preparation of the aqueous almond peel extract. All solutions were prepared with distilled water, and all chemicals were used without any further purification.

### 2.2. Methodologies

#### 2.2.1. Almond peel extract preparation.

**Fig 1:**
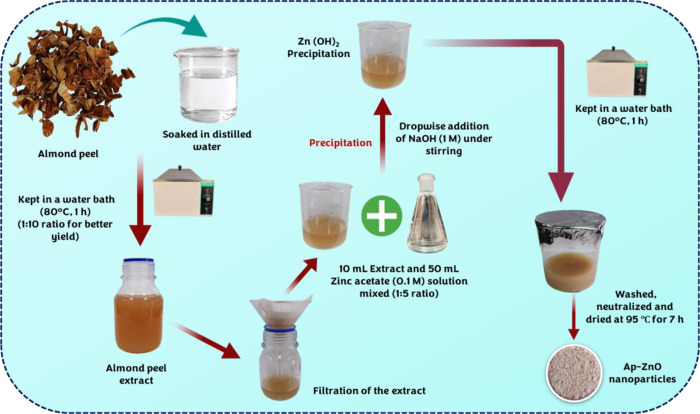
Green synthesis of Ap-ZnO nanoparticles from almond peel extract.

For extract preparation, the pretreated almond peels were mixed with distilled water (1:10). The suspension was heated in a water bath (80 ℃, 1 h) to leach out water-soluble phytochemicals. After cooling, it was filtered through Whatman filter paper to separate the liquid extract from the spent peels. The clear light brown filtrate obtained in this way, hereafter referred to as almond peel extract was used for the subsequent preparation of almond peel-derived ZnO (Ap-ZnO) nanoparticles. The sequence of steps involved in extract preparation is depicted schematically in Fig 1.

#### 2.2.2. Green synthesis of Ap-ZnO nanoparticles.

Almond peel extract and zinc acetate solution (50 mL, 0.1 M) were mixed in a 1:5 (v/v) ratio under continuous magnetic stirring. Sodium hydroxide solution (10 mL, 1 M) was added slowly to the mixture, which generated a pale white precipitate attributed to zinc hydroxide intermediates. The resulting suspension was maintained in a water bath (80 °C, 1 h), allowed to cool, and the solid contents were centrifuged (5000 rpm, 10 min). The precipitate was washed repeatedly with distilled water until the supernatant was nearly neutral and then dried in a hot air oven (95 ℃, 7 h). The dried solid was gently ground in a clean mortar to obtain a uniform Ap-ZnO nanoparticle powder that was used for all physicochemical characterization and photocatalytic experiments. The schematic illustration of the green synthesis of Ap-ZnO nanoparticles using almond peel extract is shown in Fig 1.

#### 2.2.3. Ap-ZnO nanoparticles characterization.

The optical absorption characteristics of the almond peel extract and Ap-ZnO nanoparticles were recorded using a UV-visible spectrophotometer (UV 1900i, Shimadzu, Japan). The surface texture of the Ap-ZnO nanoparticles was studied with a field-emission scanning electron microscope (FESEM, Neon 40, Carl Zeiss, Germany), and the associated elemental nature was obtained using an energy-dispersive X-ray spectroscopy system (EDS, Oxford Instruments, United Kingdom). The crystalline phase of the photocatalyst was characterized with X-ray diffraction (XRD, D8 Advance, Bruker, Germany). Fourier transform infrared (FTIR, 8400S, Shimadzu, Japan) spectra were collected to identify vibrational bands related to the ZnO lattice and almond peel-derived organics. Surface chemical states of zinc, oxygen and carbon were studied with X-ray photoelectron spectroscopy (XPS, K α, Thermo Fisher Scientific, United Kingdom). Textural properties were determined from nitrogen adsorption-desorption isotherms measured on a surface area analyser (Smart Instruments, Mumbai, India), and the specific surface area was evaluated using the Brunauer-Emmett-Teller method. Photoluminescence (PL) spectra of the Ap-ZnO nanoparticles were acquired at room temperature with a fluorescence spectrometer (V 730, JASCO, Japan) to probe near-band-edge emission (NBE) and defect-related visible bands relevant to charge-carrier recombination during photocatalysis.

#### 2.2.4. Photocatalytic experiments for CR degradation.

The photocatalytic potential of Ap-ZnO towards CR degradation was evaluated under natural sunlight. CR dye stock solution was prepared in distilled water, and suitable dilutions were made to obtain working solutions with an initial dye concentration of 25 mg/L. For each experiment, 100 mL of CR solution were placed in a 250 mL borosilicate beaker, and Ap-ZnO nanoparticles were added to obtain dosages of 5, 10 and 15 mg/100 mL of solution. All the experiments were performed at the native pH of the CR solution without any adjustment.

Before illumination, the contents were magnetically mixed at 150 rpm in dark (30 min) to approach adsorption-desorption equilibrium and to ensure uniform dispersion of the Ap-ZnO particles. The beakers were then exposed to direct sunlight with continuous stirring at 150 rpm to keep the Ap-ZnO particles uniformly suspended and maintain effective contact between the catalyst and the dye solution. Experiments were conducted on clear days between late morning and mid-afternoon in Manipal, India, during a period of relatively stable solar intensity. At regular time durations, aliquots were taken from the contents, immediately centrifuged to remove the solid photocatalyst, and the clear supernatant was analysed by UV- visible spectroscopy. The residual CR concentration at each time was determined from the absorbance at 497 nm using a calibration curve constructed from standard dye solutions. For each catalyst dose, the photocatalytic runs were done in triplicates, and the average concentration was used for further calculations. The percentage degradation of CR at time t was obtained from Eq. (1):


$$\text{Degradation }(\%)=\frac{C_{\text{0}}-C_{t}}{C_{\text{0}}}\times \text{1}\text{0}\text{0}$$ (1)

where $C_{\text{0}}\;$ denotes the initial dye concentration and $C_{t}\;$ is the concentration at time t.

The photocatalytic degradation kinetics were interpreted by a pseudo-first-order (PFO) model expressed in Eq. (2):


$$\text{ln}\left(\frac{C_{\text{0}}}{C_{t}}\right)=kt$$ (2)

where k is the apparent rate constant for CR degradation under sunlight and t is the irradiation time. The values of k for the different Ap-ZnO doses were obtained from the slope of the linear plots between $\text{ln}(C_{\text{0}}/C_{t})\;$ and t.

Reactive species trapping experiments were carried out to identify the principal active species involved in the sunlight-driven degradation of CR, following a modified procedure adapted from an earlier reported method [31]. In brief, 15 mg of Ap-ZnO was dispersed in 100 mL of CR solution having an initial concentration of 25 mg/L, and the suspension was stirred in the dark for 30 min to establish adsorption-desorption equilibrium. After this step, Disodium ethylenediaminetetraacetate (EDTA-2Na, 5 mM), Isopropanol (IPA, 10 mM), and p-benzoquinone (PBQ, 1 mM) were added separately as scavengers for photogenerated holes (h^+^), hydroxyl radicals (•OH), and superoxide radicals (•O_2_^−^), respectively. The reaction mixtures were then exposed to natural sunlight under continuous stirring. At the end of the irradiation period (270 min), aliquots were withdrawn, centrifuged to separate the photocatalyst, and the clear supernatant was analyzed using a UV-visible spectrophotometer to determine the residual CR dye concentration at 497 nm. A control experiment performed under identical conditions without the addition of any scavenger was used for comparison.

### 2.3. Reusability experiments

The reusability of Ap-ZnO nanoparticles was assessed under the optimized photocatalytic conditions using a modified procedure adapted from a previously reported method [32]. In each cycle, 15 mg of Ap-ZnO was dispersed in 100 mL of CR solution with an initial concentration of 25 mg/L and then exposed to natural sunlight. At the end of each run, the photocatalyst was recovered by centrifugation, washed successively with distilled water, ethanol, and again with distilled water, and then dried for 12 h before being reused in the subsequent cycle. The retained photocatalytic activity relative to the first cycle was used to evaluate the stability and reuse potential of the synthesized photocatalyst.

## 3. Results and discussion

### 3.1. UV-visible spectroscopy results

**Fig 2:**
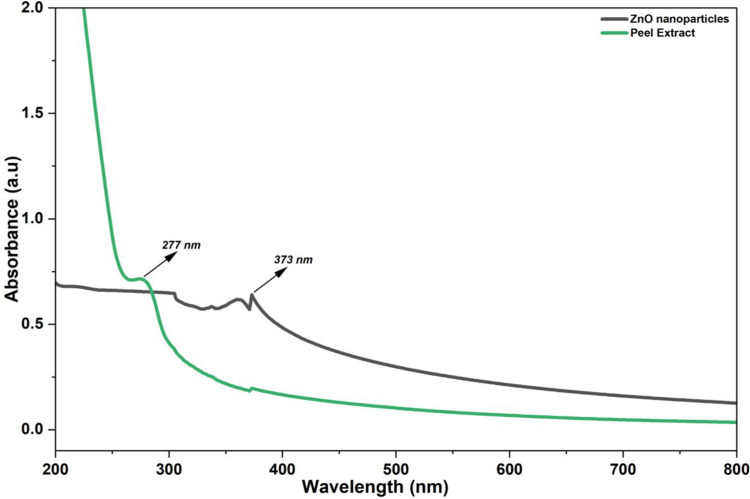
UV-visible absorption spectra of almond peel extract and Ap-ZnO nanoparticles.

The optical response of the almond peel extract and the Ap-ZnO nanoparticles was examined using UV-visible spectroscopic images (Fig 2). The peel extract spectrum shows a steep decrease in absorbance from the deep UV region toward longer wavelengths with a distinct band centred at 277 nm. This band arises from π–π* and n–π* transitions in aromatic and conjugated moieties present in phenolic and flavonoid compounds of the almond peel. Comparable UV bands at about 273 and 280 nm have been reported for *Salvadora persica* [33] and *Rubus fairholmianus* root extracts [34]. The strong absorption of the crude extract in the UV-region supports its role as a rich source of bioactive molecules that can complex zinc ions, reduce them and subsequently cap the growing inorganic phase during the synthesis step. After reaction with zinc acetate and NaOH, the spectrum of the Ap–ZnO nanoparticles displays a different profile (Fig 2). The extract band is largely suppressed, and a new broad absorption feature appears in the near UV-region at 373 nm, which is characteristic of the fundamental absorption edge of crystalline ZnO. This observation is in line with earlier reports on ZnO synthesized using *Deverra tortuosa* (374 nm) [35], *Syzygium cordatum* (375 nm) [36], and *Zingiber officinale* (376 nm) [37] extracts. Additionally, the optical band gap of the Ap-ZnO nanoparticles is estimated to be about 3.32 eV from the relation E_g_ = 1240/λ. This value closely matches with bulk ZnO band gap of (3.37 eV) [38] and is comparable to the band gap of 3.40 eV reported for ZnO nanoparticles prepared from sunflower seed husk extract [39]. The gradual tail of absorbance that extends into the visible region implies the existence of lattice defects and surface states, which are commonly connected with oxygen vacancies or zinc interstitials [40]. Such defect-related states can facilitate absorption of a larger fraction of the solar spectrum, promote separation of photogenerated charge carriers [41] and are therefore beneficial for sunlight-driven degradation of CR in aqueous solution.

### 3.2. Surface morphology and elemental composition of Ap-ZnO NPs

**Fig 3:**
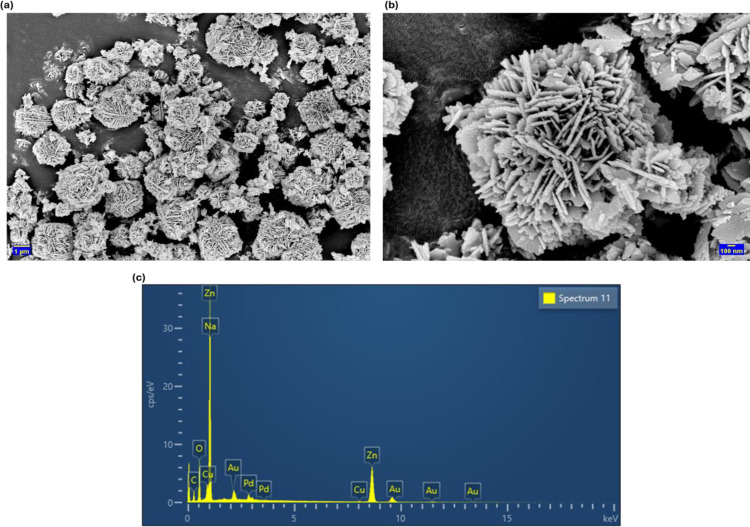
(a) Low magnification and (b) high magnification FESEM images, and (c) corresponding EDS spectrum of Ap-ZnO nanoparticles. EDS was used to probe the elemental composition of the Ap-ZnO NPs.

The low magnification image in Fig 3a shows densely packed flower-like structures. Each structure is constructed from many radially arranged plates and rods, which gives a clear flower-like architecture rather than isolated crystallites. Similar multibranched flower-like ZnO aggregates have been reported for nanoparticles synthesized by a hydrothermal route [28]. The high magnification micrograph (Fig 3b) resolves the building blocks as slender ZnO rods that grow outward from a compact central core. This hierarchical assembly suggests that numerous nuclei first formed during the reaction between zinc acetate and the almond peel extract, and that the phytochemical constituents subsequently directed anisotropic crystal growth along preferred directions [42]. Such three-dimensional flower-like structures are expected to provide a high density of exposed edges, corners and interparticle voids, creating abundant accessible sites for CR adsorption and photocatalytic reaction under sunlight.

The spectrum displays dominant signals assigned to Zn and O (Fig 3c), confirming that the products consist mainly of ZnO [43]. A very intense peak in the low energy region arises from overlap of the Zn L and Na K lines, indicating Zn together with trace Na residue from the NaOH used in precipitation. A second strong Zn peak is observed around 8–9 keV and arises from Zn K emission, which further supports the formation of ZnO [44]. The elements C and O at lower energies are associated with the surface-bound organic species derived from the extract that may remain attached to the particle surface after drying. Weak signals from Cu, Au and Pd originate from the sample holder and conductive coating used for FESEM analysis. No additional peaks due to other metallic species are detected, which implies that the green synthesis route produces ZnO with good chemical purity.

### 3.3. BET analysis

BET analysis of the Ap-ZnO nanoparticles gave a specific surface area of 31.89 m^2^/g, with a pore volume of 0.0779 cm^3^/g. The mean pore diameter was estimated to be about 9.8 nm, which places the pores in the mesoporous range. The obtained surface area lies at the upper end of the range reported for many plant-derived ZnO systems from *Artocarpus heterophyllus* peel extract (6.6 m^2^/g) [45], *Leonotis ocymifolia* (20.3 m^2^/g) [46], and ginger extract (26.74 m^2^/g) [47]. Together with its mesoporous texture and interconnected interparticle voids, this level of surface area should be sufficient to permit efficient diffusion and adsorption of CR molecules and to supply readily accessible active sites for the photocatalytic reactions in the sunlight-driven process.

### 3.4. Crystal structure of Ap-ZnO nanoparticles

**Fig 4:**
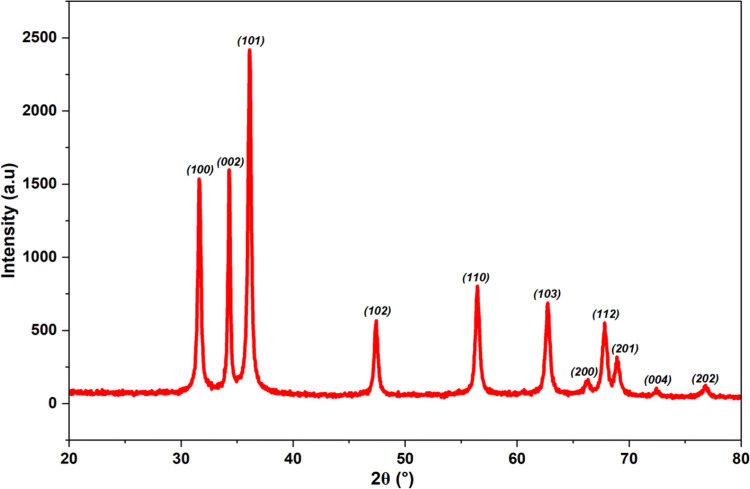
XRD pattern of Ap-ZnO nanoparticles.

The XRD image of the Ap-ZnO nanoparticles is depicted in Fig 4. A series of well-defined reflections is observed in the 2θ range from 30 to 80^ο^, with three intense peaks at about 31.6°, 34.2° and 36.1° that can be indexed to the (100), (002) and (101) planes of hexagonal wurtzite ZnO. Additional lines at approximately 47.4°, 56.4°, 62.7°, 66.2°, 67.8°, 68.9°, 72.4° and 76.7° correspond to the (102), (110), (103), (200), (112), (201), (004) and (202) planes, respectively, and match the standard data for wurtzite ZnO (JCPDS 36–1451) [48]. The close agreement of the peak positions and their relative intensities with the reference pattern is consistent with XRD profiles reported for ZnO prepared from *Epipremnum aureum* [49] and *Thymus vulgaris* [50] leaf extracts. The average crystallite size was calculated as 39.24 nm using the Scherrer equation, which is comparable to green-synthesised ZnO obtained using *Myrtus communis* extract (37.7 nm) [48] and *Dolichos lablab* leaf extract (36.07 nm) [51]. Furthermore, the interplanar spacing (d_101_) for the (101) plane is 0.248 nm, which closely matches the reported value for bare ZnO synthesized from *Bauhinia racemosa* Lam. leaf extract [52].The lattice parameters were calculated as a (=b) = 3.26 Å and c = 5.22 Å, giving a c/a ratio of about 1.60. These constants are very close to the standard parameters of bulk wurtzite ZnO [a (=b)= 3.25 Å and c = 5.20 Å; c/a = 1.60] [53], and align well with ZnO prepared from *Parthenium hysterophorus* extract [54], which indicates that incorporation of plant-derived species during synthesis does not significantly distort the ZnO lattice. No extra diffraction peaks attributable to other crystalline impurities are detected, implying that zinc is present predominantly as a single ZnO phase.

### 3.5. FTIR analysis

**Fig 5:**
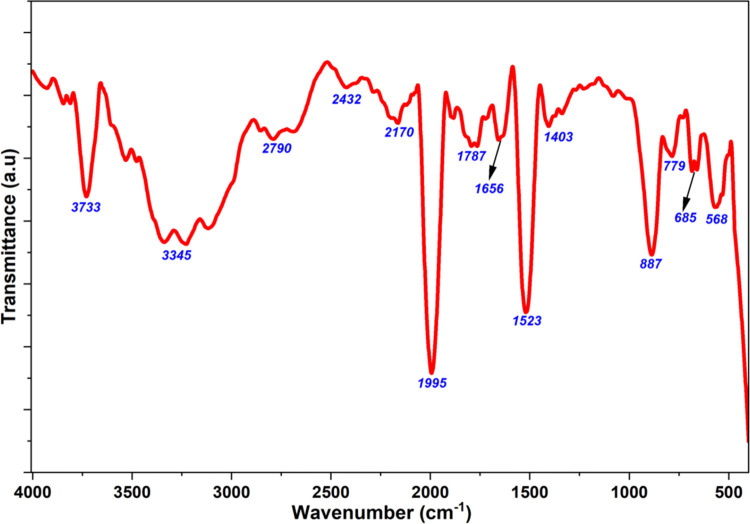
FTIR spectrum of Ap-ZnO nanoparticles.

The FTIR spectrum of the Ap-ZnO nanoparticles reveals the surface functional groups originating from both the ZnO lattice and organic species from the almond peel extract (Fig 5). In the high wavenumber region, a broad and structured band with maxima at about 3733 and 3345 cm^-1^ is observed and is assigned to O–H stretching vibrations from surface hydroxyl groups and hydrogen-bonded phenolic hydroxyl groups derived from the extract [55]. The abundance of these moieties makes the ZnO surface more hydrophilic and provides sites for photogenerated holes to form hydroxyl radicals that efficiently degrade CR under sunlight. A shoulder around 2790 cm^-1^ is related to aliphatic C–H stretching from carbohydrates, lipids, and other biomass fragments that are not fully removed during drying [54]. Weak bands near 2432 and 2170 cm^-1^ are mainly attributed to adsorbed CO_2_ [56] with minor contributions from overtone or combination bands of surface carbonyl groups.

The signals at 1995 and 1787 cm^-1^ are consistent with overtone features associated with strong C = O environment in conjugated organic species anchored to metal oxide surfaces [57]. The signal near 1656 cm^-1^ is ascribed to C = O stretching, attributable to carbonyl groups, conjugated carbonyl functionalities, and amide I vibrations [58]. The 1523 cm^-1^ signal relates mainly to aromatic C = C stretching in phenolic and flavonoid rings [59]. The 1403 cm^-1^ peak is generally associated with phenolic O–H bending [60]. In the fingerprint region, the bands at 887, 779, and 685 cm^-1^ arise from out-of-plane aromatic C–H bending modes in substituted rings, confirming that aromatic structures from the almond peel extract remain attached to the nanoparticle surface [60,61]. Finally, the distinct absorption at 568 cm^-1^ is assigned to Zn–O stretching vibrations of the wurtzite lattice, confirming the formation of crystalline ZnO as the main inorganic phase [62]. FTIR results therefore confirm that the Ap-ZnO nanoparticles consist of a crystalline ZnO phase coated with almond peel-derived organic species bearing hydroxyl, carbonyl and aromatic groups, which provide active sites for CR adsorption and enhance its photocatalytic degradation under sunlight.

### 3.6. XPS analysis of Ap-ZnO nanoparticles

**Fig 6:**
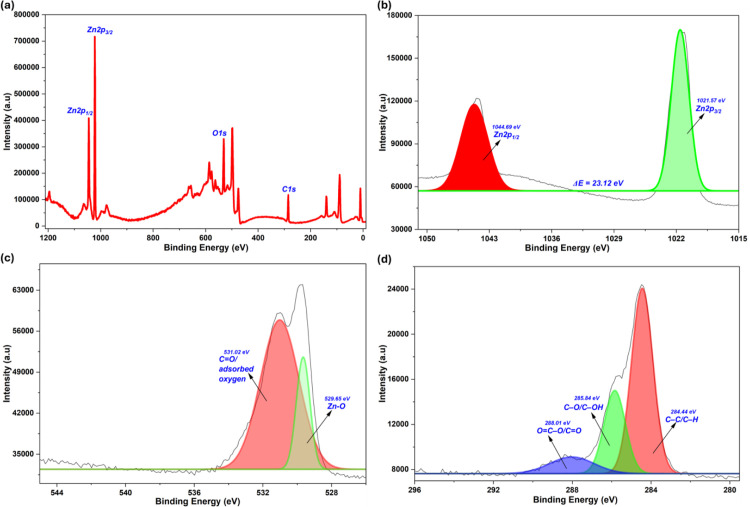
XPS spectra of Ap-ZnO nanoparticles: **(a)** survey spectrum, **(b)** Zn 2p core level, **(c)** O 1s core level, and **(d)** C 1s core level.

The wide scan spectrum (Fig 6a) shows intense features assigned to Zn 2p and O 1s, together with a weaker C 1s signal. This is consistent with the EDS and XRD results and confirms that zinc is present mainly as ZnO with a thin organic overlayer derived from the almond peel extract. Similar XPS survey spectra dominated by Zn and O with only minor C contributions have been reported for plant extract-mediated ZnO microstructures synthesized using *Cordia myxa* leaves, where the C 1s signal arises from phytomolecules adsorbed on the ZnO surface [63].

The high-resolution Zn 2p spectrum (Fig 6b) displays two symmetric signals found at 1021.57 and 1044.69 eV, which are allocated to Zn 2p_3/2_ and Zn 2p_1/2_, respectively. The energy separation between these components is 23.12 eV, consistent with the spin-orbit splitting expected for Zn^2+^ in the wurtzite ZnO lattice; a very similar difference between the Zn 2p_3/2_ and Zn 2p_1/2_ peaks has been reported for ZnO nanoparticles prepared with *Mirabilis jalapa* flower extract, where this value was taken as characteristic of Zn^2+^ in ZnO [64]. This confirms that the zinc species formed during the green synthesis are fully oxidized to ZnO and that the crystalline phase identified by XRD is also representative of the surface.

The O 1s core level (Fig 6c) can be deconvoluted into two contributions. The main component at 529.65 eV is attributed to lattice O^2-^ ions in Zn–O bonds of the ZnO framework. A second, broader component appears at about 531.02 eV and represents oxygen in surface hydroxyls, adsorbed molecular oxygen, and C = O type groups associated with the almond peel-derived organics. A similar O 1s doublet with peaks at 529.7 and 531.1 eV has been reported for plant extract-derived ZnO [65]. These oxygen-rich sites are expected to participate in photocatalytic reactions by trapping photogenerated electrons, forming superoxide or hydroxyl radicals, and thereby complement the defect-related states inferred from the UV visible tail.

The C 1s spectrum (Fig 6d) further supports the presence of an organic coating from the extract. Deconvolution yields three main components. The dominant peak at 284.44 eV corresponds to C–C and C–H bonds in aliphatic and aromatic carbon. A second contribution at 285.84 eV is assigned to C–O or C–OH species, while a higher binding energy peak at 288.01 eV arises from O–C = O or C = O groups. These functionalities agree well with the hydroxyl, carbonyl, and aromatic bands observed in the FTIR spectrum, and confirm that phytochemicals from the almond peel remain anchored on the ZnO surface. A very similar set of C 1s components attributed to C–C, C = O and O–C = O environments has been reported for ZnO nanoparticles prepared from neem plant leaves [66]. These oxygen-containing carbon species offer extra binding sites for CR adsorption through hydrogen bonding and pi-pi stacking and also promote interfacial electron transfer, which enhances ROS formation during sunlight-driven photocatalysis.

### 3.7. Photoluminescence characteristics of Ap-ZnO nanoparticles

**Fig 7:**
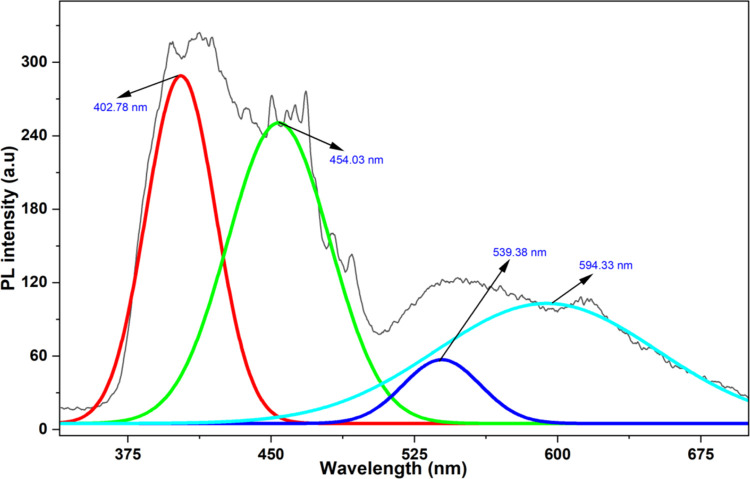
Photoluminescence (PL) spectrum of Ap-ZnO nanoparticles with Gaussian deconvolution of emission bands.

The room temperature PL spectrum of the Ap-ZnO nanoparticles recorded under UV excitation is shown in Fig 7. The experimental profile is broad and asymmetric, extending from the near UV into the visible region. There are four distinct emission bands with maxima at about 402.78, 454.03, 539.38 and 594.33 nm. The first component in the violet region near 402.78 nm (3.07 eV) is assigned to NBE excitonic recombination in crystalline ZnO [67]. This agreement between the absorption edge and the NBE emission confirms that the nanoparticles retain the wurtzite ZnO electronic structure identified by XRD. The band centred at 454.03 nm (2.73 eV) lies in the blue region and is generally associated with shallow defect states such as zinc interstitials or zinc vacancy related donors that participate in radiative recombination [68]. The green emission around 539.38 nm (2.29 eV) and the orange-red band near 594.33 nm (2.08 eV) are characteristic of deeper defects including singly ionized oxygen vacancies (V_o_), oxygen interstitials and surface-related states [69]. The presence of these visible bands is in line with the absorption tail that extends into the visible region in the UV-visible spectrum and with the oxygen-rich surface species revealed by FTIR and XPS analyses. These results indicate that the Ap-ZnO nanoparticles contain a significant population of lattice and surface defects together with chemisorbed oxygen and almond peel-derived functional groups on the surface. Such trap states are beneficial for sunlight-driven CR degradation, since they can slow down direct electron-hole recombination and favour the formation of ROS through interfacial charge transfer to adsorbed oxygen or surface hydroxyl groups [67]. The PL response, therefore, supports the suitability of Ap-ZnO nanoparticles as an efficient photocatalyst for CR removal under natural sunlight.

### 3.8. Sunlight-driven CR dye degradation

**Fig 8:**
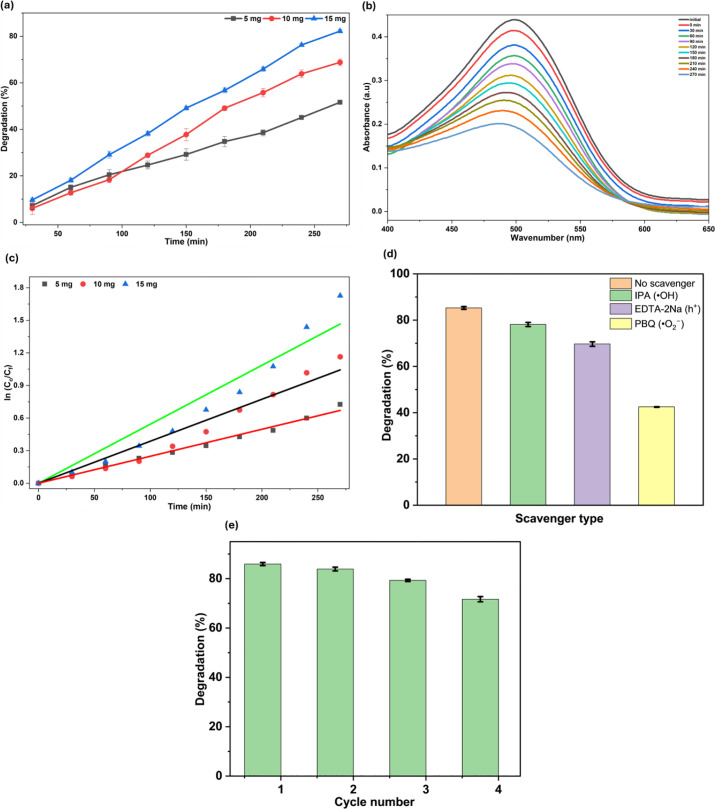
(a) Photocatalytic degradation of CR under sunlight at different Ap-ZnO dosages, (b) time-dependent UV visible absorption spectra during degradation, (c) pseudo-first-order kinetic plots for CR degradation, (d) effect of different radical scavengers on the sunlight-driven degradation of CR over Ap-ZnO, and (e) reusability performance of Ap-ZnO during successive photocatalytic cycles.

The photocatalytic potential of the Ap-ZnO nanoparticles toward CR removal under natural sunlight was evaluated by monitoring the temporal change in dye concentration at different catalyst loadings (Fig 8a). For all three catalyst doses, the degradation efficiency increased steadily with irradiation time, showing that the contact between the catalyst and the dye solution remained effective throughout the 270 min experiment. The highest removal was obtained for 15 mg of Ap-ZnO (82.21%), followed by 10 mg (68.79%) and then 5 mg (51.61%). The strong dependence of degradation efficiency on catalyst dose reflects the larger number of active surface sites and light-absorbing centres available at higher Ap-ZnO loadings, which facilitates adsorption of CR molecules and generation of ROS during sunlight exposure [70].

Further insight into the degradation process can be obtained from the time-resolved absorption spectra of CR recorded for the system containing 25 mg/L dye and 5 mg of Ap-ZnO (Fig 8b). The initial solution shows a pronounced absorption band in the visible region at 497 nm that is characteristic of the azo chromophore of CR dye. As irradiation proceeds, this band steadily loses intensity, reflecting the gradual decrease in CR concentration as dye molecules interact with Ap-ZnO and are degraded under sunlight. A similar time-dependent decay of the visible absorption band has been reported for methylene blue and malachite green solutions treated with ZnO nanoparticles synthesized using *Ruellia tuberosa* leaf extract [71].

The kinetic data were interpreted using a PFO model commonly employed for heterogeneous photocatalysis. For all three catalyst doses, the plots of ln (C_0_/C_t_) against irradiation time were nearly linear, with R^2^ of 0.996 (5 mg), 0.979 (10 mg) and 0.974 (15 mg) (Fig 8c), confirming that CR degradation can be described by PFO kinetics. Comparable PFO behaviour has been reported for other green synthesised ZnO systems, including ZnO obtained from *Thymus vulgaris* leaf extract for the degradation of methylene blue and Sunfix red [50], and ZnO prepared using para rubber leaf extract for methylene blue [72]. The apparent rate constants obtained from the slopes were 0.0025, 0.0039 and 0.0054 min^-1^ for 5, 10 and 15 mg of Ap ZnO, respectively. The systematic increase in the rate constant with catalyst dosage demonstrates that higher Ap-ZnO loadings accelerate CR degradation, consistent with a larger number of illuminated active sites, more efficient utilization of sunlight and enhanced generation of ROS at the catalyst surface [15].

To further clarify the active species responsible for sunlight-driven CR degradation over Ap-ZnO, reactive species trapping experiments were performed under the optimized conditions and the results are presented in Fig 8d. In the absence of any scavenger, the degradation efficiency reached 85.27%. Upon addition of IPA, the degradation decreased slightly to 78.14%, indicating that •OH radicals participated in the reaction but were not the dominant oxidizing species. A stronger suppression was observed in the presence of EDTA-2Na, where the degradation dropped to 69.69%, showing that photogenerated h^+^ also made a significant contribution to CR oxidation. The most pronounced inhibition occurred after adding PBQ, for which the degradation efficiency fell sharply to 42.48%. This substantial decline demonstrates that O_2_•^−^ radicals were the principal reactive species governing the photocatalytic process. A similar scavenger trend has been reported in earlier dye photocatalysis studies. Wang *et al.* found that IPA caused only a slight decrease in degradation, whereas EDTA-2Na and PBQ produced much stronger inhibition, indicating that h^+^ and O_2_•^−^ were the dominant reactive species [73]. Consistent with this, Vázquez Dávila *et al.* observed only a minor effect with IPA but marked suppression in the presence of PBQ and EDTA-2Na, further supporting the dominant role of O_2_•^−^ and h^+^, while •OH made a smaller contribution [74].

### 3.9. Proposed mechanism for sunlight-driven degradation of CR dye

**Fig 9:**
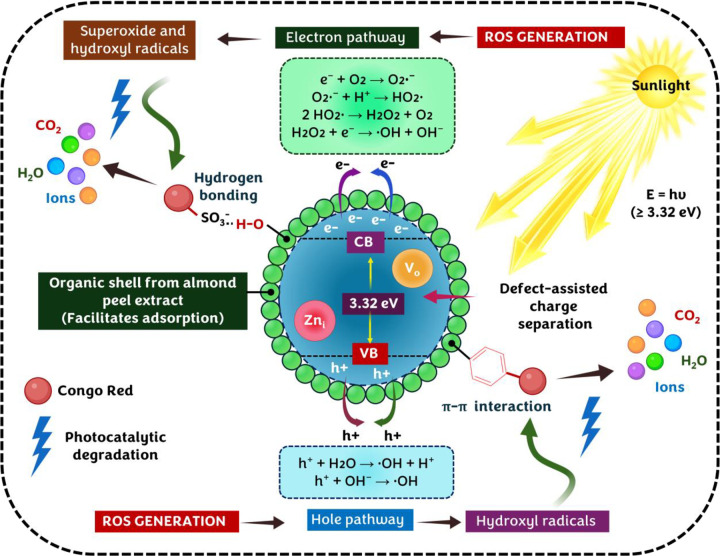
Proposed mechanism for sunlight-driven photocatalytic degradation of CR dye over Ap-ZnO nanoparticles (Created using BioRender.com).

The photocatalytic behaviour of Ap-ZnO toward CR dye (Fig 9) can be rationalized by considering the electronic structure of ZnO, the defect states, and the organic shell introduced by the almond peel extract. UV visible measurements show an absorption edge near 373 nm with an optical band gap of about 3.32 eV together with a tail extending into the visible region. This tail, together with the blue and visible PL bands, points to a significant density of shallow and deep defect states associated with zinc interstitials (Zn_i_) and oxygen vacancies (V_o_) (Fig. 9) [75]. These centres, distributed throughout the wurtzite lattice and at the surface, act as traps for photogenerated carriers and help to decouple electron and hole migration paths under sunlight.

The flower-like assemblies observed in FESEM consist of radially arranged ZnO rods packed into spheroidal secondary particles. This hierarchical morphology, combined with the mesoporous texture and specific surface area of 31.89 m^2^/g, creates a network of edges, corners and interparticle voids that can host CR molecules close to the active semiconductor domains. FTIR and XPS analyses show that the ZnO surface is coated by almond peel-derived species carrying hydroxyl, carbonyl and aromatic groups, together with surface hydroxyls and chemisorbed oxygen. These functionalities may provide multiple interaction modes for the CR dye. The sulfonate groups of CR may interact electrostatically with protonated ZnO surface sites and form hydrogen bonds with the organic shell, while π–π contacts with aromatic residues further stabilize adsorption at the interface [76]. As a result, dye molecules become pre-concentrated at or near the semiconductor interface before and during illumination.

Under natural sunlight, photons with energy equal to or larger than the band gap promote electrons from the valence band (VB) to the conduction band (CB) of Ap-ZnO and leave holes (h^+^) in the valence band [77], as shown in Equation (3).


$$\text{Ap}-\text{ZnO}+\text{h}\nu \to \text{Ap}-\text{ZnO }(\overset{-}{\underset{\text{CB}}{\text{e}}}+\overset{+}{\underset{\text{VB}}{\text{h}}})$$ (3)

The defect-related states inferred from PL can act as trap centres that transiently capture a fraction of the photogenerated carriers, thereby retarding rapid electron-hole recombination. Trapped electrons migrate to the surface and react with dissolved oxygen or chemisorbed oxygen species to form superoxide radicals [78], as described in Equations (4)-(6).


$$\overset{-}{\underset{\text{CB}}{\text{e}}}+{\text{O}}_{\text{2}}\to \overset{•-}{\underset{\text{2}}{\text{O}}}$$ (4)

$$\overset{•-}{\underset{\text{2}}{\text{O}}}+{\text{H}}^{+}\to \text{H}\overset{•}{\underset{\text{2}}{\text{O}}}$$ (5)

$$\text{2H}\overset{•}{\underset{\text{2}}{\text{O}}}\to {\text{H}}_{\text{2}}{\text{O}}_{\text{2}}+{\text{O}}_{\text{2}}$$ (6)

Hydrogen peroxide (H_2_O_2_) formed *in situ* can be further reduced or photochemically converted to generate hydroxyl radicals [77], consistent with Equation (7).


$$H_{\text{2}}O_{\text{2}}+\overset{-}{\underset{CB}{e}}\to •OH+OH^{-}$$ (7)

In parallel, VB holes and surface-trapped h^+^ oxidize surface hydroxyl groups or water to additional hydroxyl radicals [22], as captured in Equations (8) and (9).


$$\overset{+}{\underset{\text{VB}}{h}}+H_{\text{2}}O\to •OH+H^{+}$$ (8)

$$\overset{+}{\underset{\text{VB}}{h}}+OH^{-}\to •OH$$ (9)

The FTIR and XPS results show abundant surface hydroxyls and oxygen-rich carbon species, which provide a dense population of sites where these reactions can occur. The organic coating from the extract may also serve as an intermediate electron donor, assisting charge transfer to oxygen and stabilizing radical intermediates at the solid-liquid interface [79]. Altogether, these processes establish a reactive shell around the Ap-ZnO particles that is rich in superoxide and hydroxyl radicals. CR molecules that are adsorbed on this surface or located in its immediate vicinity are then attacked by the ROS. The first steps usually involve cleavage of the azo linkages and opening of the aromatic rings [80], as indicated in Equation (10).


$$•OH\text{ or }\overset{•-}{\underset{\text{2}}{O}}+\text{CR}\to \text{short lived aromatic intermediates}$$ (10)

Subsequent oxidation steps convert these fragments into low molecular mass carboxylic acids and eventually to CO_2_, water and inorganic ions derived from the sulfonate and amino groups [81].

### 3.10. Reusability of Ap-ZnO nanoparticles

The reusability of Ap-ZnO nanoparticles was evaluated over four successive photocatalytic cycles under the optimized conditions, and the corresponding degradation efficiencies are presented in Fig 8e. A gradual decline in performance was observed with repeated use, with the degradation efficiency decreasing from 85.93% in the 1^st^ cycle to 83.91, 79.31, and 71.87% in the 2^nd^, 3^rd^, and 4^th^ cycles, respectively. Despite this progressive decrease, the photocatalyst retained about 83.64% of its initial activity after four cycles, indicating satisfactory stability and reuse potential under natural sunlight. The reduction in activity may be attributed to surface poisoning by degradation intermediates, which can block active sites and lower the photocatalytic performance of the catalyst, along with slight particle agglomeration during repeated use [82,83]. These results demonstrate that Ap-ZnO possesses reasonably good reusability and holds promise as an effective green photocatalyst for the treatment of dye-contaminated water.

## 4. Conclusion

This study established an environmentally benign strategy for synthesizing flower-like ZnO nanostructures using aqueous almond peel extract as the sole complexing and stabilizing medium and demonstrated their effectiveness for sunlight-activated Congo Red degradation. Spectroscopic and surface analyses further verified that phytochemical residues remain anchored on the ZnO surface as an organic overlayer enriched with oxygen-containing functional groups, together with chemisorbed oxygen species. In parallel, optical signatures indicated defect-related electronic states that can prolong charge carrier lifetimes and support interfacial redox chemistry under solar irradiation. The photocatalytic response under natural sunlight showed that dye decolorization proceeds efficiently and is well described by pseudo-first-order behaviour, consistent with a surface mediated reaction pathway. Mechanistically, the flower-like morphology promotes effective dye pre-adsorption and light harvesting, while the combined presence of surface functional groups and defect sites facilitates charge separation and accelerates ROS formation at the solid-liquid interface. Reactive species trapping confirms that the photocatalytic mechanism is primarily governed by O_2_•^−^ radicals, supported by h^+^, while •OH radicals have a negligible influence. The photocatalyst demonstrated satisfactory stability over four cycles, with only a gradual decline in efficiency, likely due to surface fouling and minor agglomeration. Tailoring phytochemical capping and defect populations, and coupling ZnO with benign supports, may further improve solar utilization and extend applicability to other organic pollutants.

## Supporting information

S1 File Minimal data set. (XLSX)
